# Accessible Real-Time Surveillance Radar System for Object Detection

**DOI:** 10.3390/s20082215

**Published:** 2020-04-14

**Authors:** Seongha Park, Yongho Kim, Kyuhwan Lee, Anthony H. Smith, James E. Dietz, Eric T. Matson

**Affiliations:** 1 Mathematics and Computer Science Division, Argonne National Laboratory, Lemont, IL 14003, USA; yongho.kim@anl.gov; 2 Institute for Information & Communications Technology Promotion, Daejeon 300010, Korea; berg76@iitp.kr; 3 Department of Computer and Information Technology, Purdue University, West Lafayette, IN 15701, USA; ahsmith@purdue.edu (A.H.S.); jedietz@purdue.edu (J.E.D.); ematson@purdue.edu (E.T.M.)

**Keywords:** accessible radar, surveillance radar, real-time object detection

## Abstract

As unmanned ground and aerial vehicles become more accessible and their usage covers a wider area of application, including for threatening purposes which can cause connected catastrophe, a surveillance system for the public places is being considered more essential to respond to those possible threats. We propose an inexpensive, lighter, safer, and smaller radar system than military-grade radar systems while keeping reasonable capability for use in monitoring public places. The paper details the iterative process on the system design and improvements with experiments to realize the system used for surveillance. The experiments show the practical use of the system and configuration for a better understanding of using the system. Cyber-physical systems for outdoor environments can benefit from the system as a sensor for sensing objects as well as monitoring.

## 1. Introduction

Unmanned aerial vehicles (UAVs), also named drones, and unmanned ground vehicles (UGVs) are explosively utilized in various circumstances such as in military, industry, and in public. The volume and weight of the unmanned vehicles vary, and specifications of each unmanned vehicle in terms of speed and payload are different per the physical features of the vehicles. The unmanned vehicles have been smaller, lighter, and, importantly, easier to control due to improvements of relevant technologies. In terms of accessibility, there has been a rapid rise in the possibility of reoccurring threats using unmanned vehicles. Within the accessibility of unmanned vehicles, operating those vehicles in public without rules and regulations rises an issue. Because of this, any person in an open public place is a possible target of a threat, and detecting the unmanned vehicle before it reaches the person to injure is a challenge that needs to be studied thoroughly.

Unfortunately, those worries and concerns of operating unmanned vehicles for threatening purposes have been realized in the last years. An unidentified unmanned vehicle was landed on the backyard of White House in Washington D.C. late night in January 2015 and left undiscovered until next morning [1]. In another case, there was an attempt of a drone attack that happened in Venezuela targeting the president of Venezuela [2]. Two drones used for the attack carried explosive materials and self-exploded in the middle of a parade above the people nearby. These drone attacks clearly showed the possibility of further drone attacks on people.

Vision-based sensors such as cameras have been popularly adopted to detect and track moving objects [3,4]. Vision information is intuitive for people when monitoring a place to find and follow an object in real-time. However, in general, monitoring by humans is costly and monitoring by computers is computationally expensive. Moreover, monitoring an open space requires monitoring systems that can perform regardless of sound noise, weather, and light conditions, or obstacles on the line of sight, because unmanned vehicles can operate on those conditions. For that matter, vision sensors or sound sensors are less robust in those aspects. To satisfy the requirements, radar technology can be adopted as a sensor to monitor public places as the technology that is robust on the environment.

To take advantage of the robustness against situational conditions, compact and accessible radar systems for area monitoring and recognizing surrounding area have been studied. Recently a surveillance UGV utilizing a radar system is proposed to monitor and explore a certain area for military purpose [5,6]. Moreover, the radar system is utilized for the research area on collision avoidance [7,8]. As shown in the previous researches, the radar system has a large potential to be utilized for monitoring and navigation.

Radar sensors that are deployed in public places need to be safe for human health. Radars that transmit high energy can cause skin burns or eye cataracts when the electromagnetic fields penetrate exposed skin surfaces or eyes [9]. Because the existing radar systems that have been utilized in the maritime navigation system, air traffic control, and weather forecasting are not affordable due to its large volume, high operation cost, and a possibility of harm to human health, the radar system we propose in this research overcomes those difficulties.

Using a radar system as a sensor is still in its early stage of being widely utilized in the research community. Therefore, the main contribution of the paper is to prototype, improve, test, and validate a compact and cost-effective mobile 2.4 GHz radar system for object detection [10,11]. The 2.4 GHz band is widely used for Wi-Fi, and the band is in the radio spectrum reserved for industrial, scientific, and medical (ISM) purposes other than telecommunications in worldwide, that is 2.4 GHz to 2.5 GHz. The band is open for amateurs along with 5.725 GHz to 5.875 GHz band. However, only the 150 MHz bandwidth is allowed in the 5.8 GHz band, which is narrower than 1 GHz bandwidth of ISM band. For radar system, wide bandwidth is engaged to dense distance resolution, therefore wide bandwidth is appropriate for a radar system. The system is designed to be deployed in a certain public area to provide monitoring for a certain height and distant range [12]. The work is motivated by the works in [13,14] as the radar systems are designed as a small, low-cost, and low-power radar system. The development for the radar system includes hardware implementation of the system in terms of improving the performance of the radar and making the system printable into a board to produce the system compact and mobile, and the software components of the system in terms of data collection and transmission between data collection device and postprocessing device. The system is demonstrated in a circumstance where a single target is moving in the sight of the radar. To test and validate the system, we set targets as a car, person, and a class 1 UAV.

## 2. Related Work

Radar technologies and applications using pulse radar have been developed in accordance with the development of weapon systems in both ground and air [15,16]. One of the purposes for the development of radar systems was to discover and identify the military strength of the opponent side in terms of ground fire and position of the military forces [17]. Targets for radar systems have been expanded and diverse as radar technologies using frequency-modulated continuous-wave (FMCW) technique has been developed and started being implemented for sensor applications.

One universal usage of the FMCW radar system is synthetic aperture radar (SAR) and inverse synthetic aperture radar (ISAR). Both of these radar systems have been particularly utilized in surveillance and reconnaissance for surrounding environments. For the reconnaissance and mapping environment, SAR and ISAR have been performing its work in the sky and on the ground, respectively. Both radar systems, SAR and ISAR, are the same in terms of engineering and science supporting the systems; nevertheless, they are different in their target and relationship of the system to the target.

With the decent development and improvement of a compact and low-power FMCW radar, many radar systems have been developed [10,11,13,14]. Those small and compact radar systems are utilized for real-time navigation and surveillance purposes as data collection and processing are now happening in real-time [18]. The radar systems also have the potential to be used as a sensor for auto-pilot and auto-driving. Moreover, the ability to see a dense object behind the wispy obstacles and easy distinguishability of open and blocked area makes the radar systems one appropriate tool for reconnaissance missions [5,19] and monitoring rural or urban area [20] in research, military, and industrial fields, along with small unmanned vehicles.

Unmanned vehicles provide mobility and maneuverability to the radar sensors to operate in a broad area in applications. Those applications include mapping inside of a building or a flat area, collecting data, imaging areas of fire or disaster, relaying communication, and searching and rescuing people in disaster areas. In particular, applications for traffic surveillance, public safety, and performing scientific research such as Earth sensing reconnaissance and scientific data collection purposes have actively adopted UAVs [21,22,23,24]. The applications have been evolved with respect to improvement of mission safety, flight repeatability due to improving auto pilots, and reduced operational cost compared to a manned aircraft.

Class 1 UAVs with accordance with the UAV classification of NATO [25] have been actively utilized in research fields because they can operate close to the ground, around various obstacles, and indoor environment [26]. Moreover, the micro UAVs can fly along hallways carrying sensors such as inertial measurement units (IMUs), camera [27], and transmitters and receivers for communication [28], while remotely communicating with the control station. With the advantages, SAR systems loaded on a fixed-wing manned vehicle and UAVs to perform missions such as Earth observation [29], monitoring and reconnaissance urban area using imaging system have been studied [30,31]. Furthermore, the radar systems can be used for simultaneous localization and mapping (SLAM) [32,33].

The combination of the radar system and unmanned vehicle is a popular research topic because of its possibility of expanding the area of application. In this research, we designed, implemented, and tested a radar system to detect a single target to show the accessibility of this system to further applications. To make it possible to utilize this system in public, the system is designed to satisfy the requirements for safety issues that are explained above. Moreover, the system is not only targeting big objects such as a car and a person, but also targeting class 1 UAVs which are popularly adopted to people and maneuvered for recreational purposes.

## 3. Development of Short-Range Object Detector

**Figure 1:**
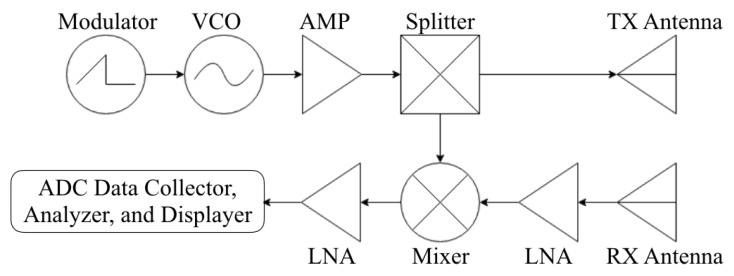
Basic frequency-modulated continuous-wave (FMCW) radar components.

The proposed system adopts basic FMCW radar system design as shown in Figure 1, which consists of several components as follows. A modulator that generates voltage within a certain voltage range with a certain interval; a voltage-controlled oscillator (VCO) that generates frequency according to the input voltage generated from the modulator; a splitter that splits the signal generated from VCO and sends the signal to both a transmit (TX) antenna and a mixer; two low-noise amplifiers (LNAs), respectively; placed in each side of the mixer that each amplifies the signal coming from the previous stage; a mixer that mixes the amplified reflected signal with the transmitted signal received from the splitter; and two directional antennas to transmit and receive signal.

### 3.1. Initial System

**Figure 2:**
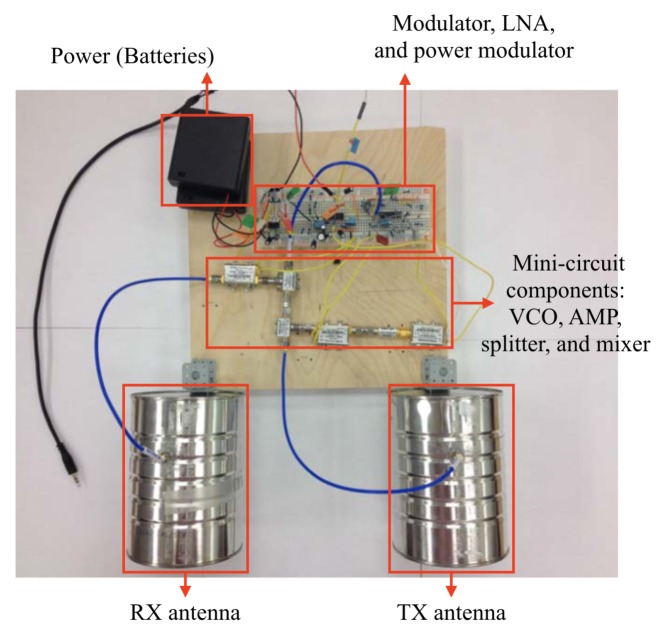
Original hardware design of the radar system (version zero).

The system we initially developed is referenced from the design of the previous studies aimed at developing low-cost and low-power radar system [13,14]. The design of the first hardware follows the original design and configuration of MIT Cantenna as shown in Figure 2. In this version, the commercialized radio components from “Mini-circuit” are used and circuits for post-signal-processing are temporally implemented on a breadboard. The components from the power are supplied by 12 V DC consisting of eight 1.5 V batteries. The transmission power of the system is approximately 25 mW.

The system utilizes two coffee cans for each TX and RX antenna. Each can has a copper wire as a wave probe installed inside of the can in order to use the can as an antenna, as proposed in the previous study [13]. With a 100 mm diameter and 132 mm length tin can, the length of the probe needs to be 44.452 mm and the location of the probe needs to be 30.776 mm from the bottom of the can. The cut-off frequency is 1.758 GHz which lower than the operating frequency of the system, which is 2.30 GHz to 2.40 GHz. The specifications of can-cantennas that we used in version zero radar are shown in Table 1.

This system does not support real-time processing at this moment because no further computation is attached to the output of the system. Instead, an audio input port on a laptop is connected to the output to collect data. The internal sound card on the laptop converts incoming data from the port into a sound file to process and obtain result later. This postprocessing supports analog–digital conversion with 16-bit resolution and 44,100 sampling rate as typical sound recording on a laptop supports.

### 3.2. Improved System

**Figure 3:**
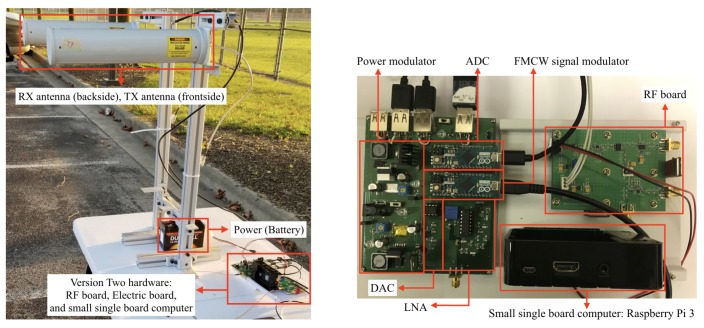
Version two hardware including antennas (**left**). Version two hardware of electric parts (**right**).

**Figure 4:**
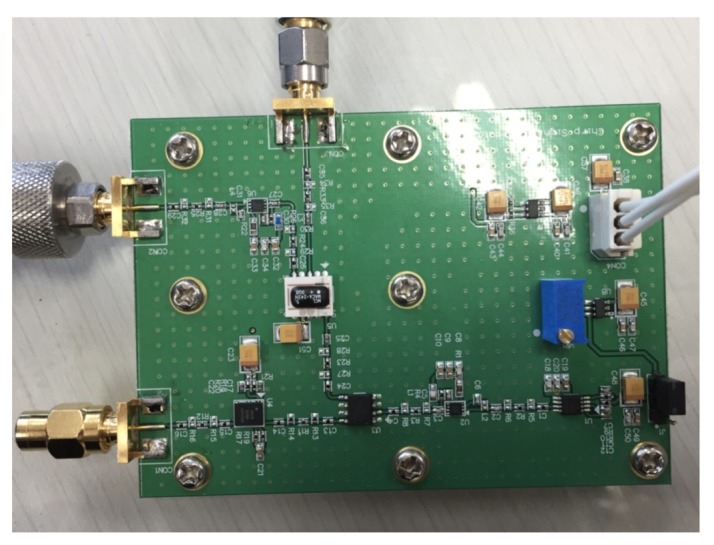
Assembled chirp signals generator; first version of RF board.

**Figure 5:**
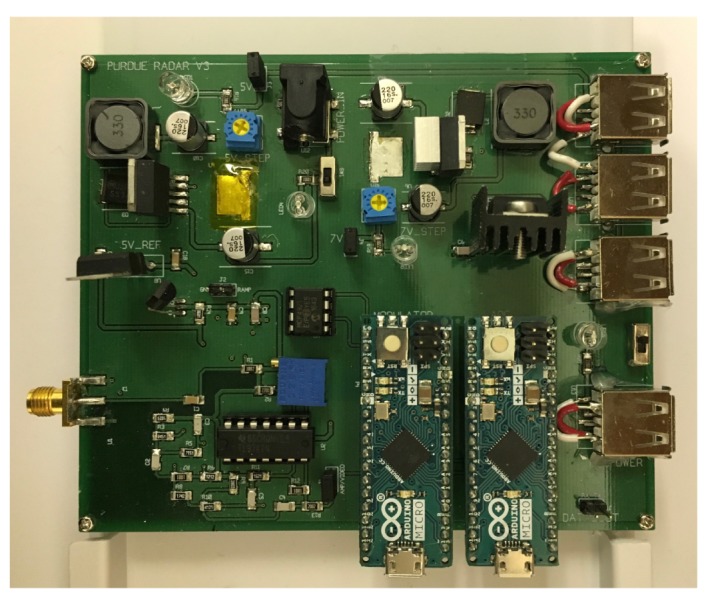
The second version of printed circuit board (PCB) for electronic parts.

The system is further improved from the previous system design by compacting the electronic components, and by fabricating the RF components into a single RF board. Figure 3 shows the overview of the hardware components in this version, and Figure 4 shows a closer look at the RF board. The RF board supports higher power transmission than the off-the-shelf components for better performance. In advance, we implement power supply, voltage regulator, and power distribution parts support the power consuming components in the single printed circuit board (PCB) as shown in Figure 5. We design to power all the analog signal processing components individually to reduce any noise from both the power circuit and other components sharing the power. Moreover, we used a micro-controller board “Arduino Micro” as an analog–digital converter (ADC) and a single-board computer, Raspberry Pi 3 are implemented in the system to provide data pipeline for real-time data analysis. In accordance capability of the Arduino board utilized as ADC, the sampling rate of the system is reduced. We adopted commercialized antennas for this improved system which can be operated in the ISM band and provide higher gain as shown in Table 2. This will be explained more in following section.

The RF board, “Chirp signals Generator (CSG) V1.0”, generates chirp signals with regard to the input voltage that is sent from the modulator. The CSG board generates signals in industrial, scientific and medical (ISM) radio band, which is the frequency band of 2.4 GHz to 2.5 GHz. The supply voltage of the board is 5 VDC and the board supports two modes: single radar frequency and frequency in range with accordance of the input ramp voltage. When the system generates single radar frequency, no other input voltage signal is required, and a circuit in the CSG board generates necessary voltage for the oscillator. The blue squared potentiometer shown in Figure 4 can be adjusted to change the input voltage for single frequency generation mode. However, to generate frequency-modulated continuous-wave, the input ramp voltage from outside is required.

The RF board is designed to generate ~1 Watt of transmission signal to gain further distance, as compared to the previous system. Comparison of the radar versions in terms of radio characteristics are shown in Table 3. The board generates lots of heat due to the intense transmission power. To reduce heat, the RF board is attached to a passive heat sink.

In addition to the CSG, the modulator and power distribution parts are also designed and fabricated in a PCB board as shown in Figure 5. The modulator is also replaced to a micro-controller board “Arduino Micro”. By adopting the Arduino board, it becomes easier to adjust ramp signals using the ability of generating digital signals ranged from 0 to 5 VDC. A digital–analog converter (DAC) circuit that is also used in the system of UCD [14] is added beside the Arduino board because the signal generated from the Arduino board is digital, but the VCO requires analog signals. The DAC is embedded on the PCB board and the Arduino board is directly wired with the DAC chip.

Furthermore, the design of LNA and power distribution parts are designed and implemented in the same PCB board on which the modulator is embedded to make the hardware of the system compact. The circuit of the LNA is the same as the previous version, except that the IC chip is replaced to another chip with similar specification simply because the IC chip was discontinued. The output of the LNA board is now collected from another Arduino Micro in real-time. With it, the radar system is no longer dependent on a laptop. The Arduino Micro is directly wired to the LNA board and performs the data collection part. The collected data are then sent to a small single-board computer, Raspberry Pi 3. The single-board computer is implemented for data processing and population. This makes the system portable and possible to be embedded in unmanned vehicles as a sensor.

The power for each device, such as the RF board, the Arduinos, and Raspberry Pi, is supplied through a power distribution circuit. Two-step voltage regulators, two linear voltage regulators, and a voltage regulator transistor are installed on the board as shown in Figure 5. The total power consumption of the RF board is approximately 7 W input, and the total power consumption of other parts is ~1.08 W. A series of step and linear regulators are allocated only for the RF board to minimize noise from the other components to the power circuits. A 12 VDC battery is employed as a power supply, and the power is regulated with regard to each of the subsystems requires. The LNA and DAC are provided 5 VDC and 2.5 VDC as a reference to the DAC chip. Two switches are added on the board to control power flow of the whole circuit board and the RF board.

## 4. Development of Real-Time Data Processing Pipeline

**Figure 6:**
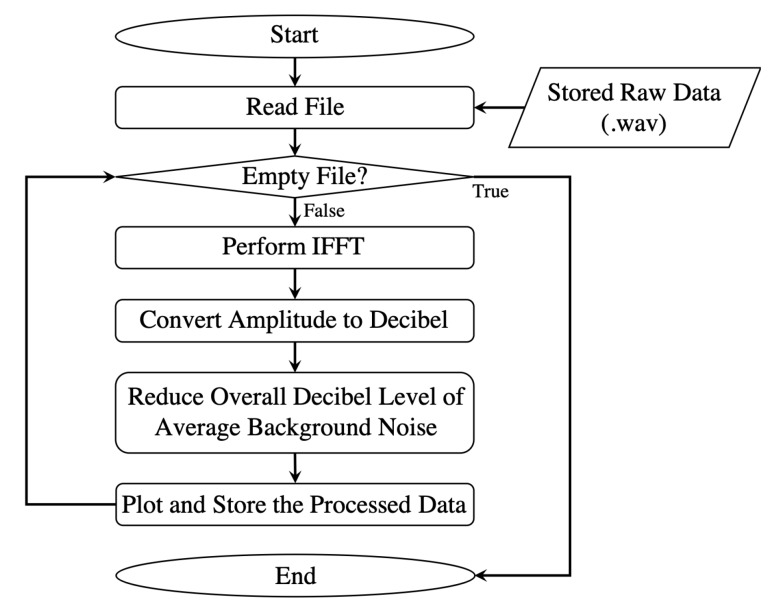
Data processing flow for the first version of the radar system.

Data processing in the initial version is performed separately from the data collection described in the Section 3.1. As shown in Figure 6, the stored raw data is a wave file that contains raw readings of the reflected signal of an object converted from the analog–digital converter. This discrete data points are fed into inverse fast Fourier transformation (IFFT) process to be converted into amplitude in the frequency domain. After that, the result of the process is accumulated into range-decibel in time domain and plotted. If the wave file is empty or all the data in a wave file are processed, the postprocessing procedure is terminated.

**Figure 7:**
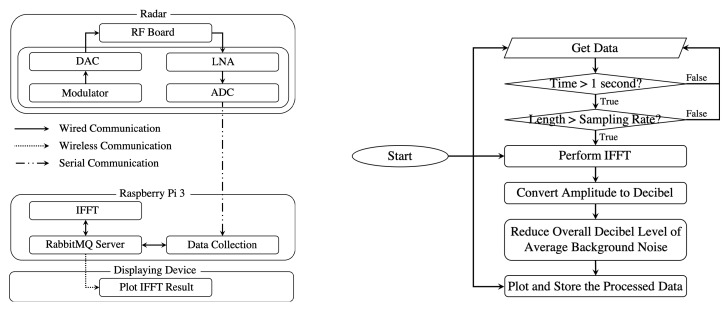
Component diagram for version two radar system (**left**), data processing flow for the version two radar system (**right**).

As the radar becomes mobile and portable by implementing circuits and small computers, it is necessary to collect and transfer raw data through wireless network communication. The hardware configuration of the radar system that has mobility is composed of the radar, a Raspberry Pi 3, and a displaying device as shown in Figure 7 (left). When the system is connected to Raspberry Pi 3, the radar can be connected to another device through the Wi-Fi connection of the Raspberry Pi 3 board for displaying results or further data processing in different devices.

As a method to connect and transmit data in real-time between the devices, one of the advanced message queuing protocol (AMQP)s named “RabbitMQ” [34,35] is adopted. RabbitMQ is an open source advanced message queuing protocols. It provides a service that delivers messages between publishers and clients. This predefined messaging protocol allows the proposed system to process and deliver data in parallel and real-time. In the system, the Raspberry Pi 3 is the server that distributes collected data. Thus, all the data are queued in the server and delivered to clients, such as the IFFT processing module inside the Raspberry Pi 3 and the result displaying script in the displaying device. Each queue has its routing key that allows postprocessing script to subscribe with accordance of what it requires to receive.

By adopting an Arduino Micro board for data collecting and passing them to Raspberry Pi 3, its sampling rate cannot reach 44,100 due to the limit of processing power. To fit the sampling rate that Arduino can support, its sampling rate is reduced to 5862, which is the least maximum samples that the Arduino can collect. With the sampling rate, the range resolution is approximately 0.37 m, and the maximum detection range is approximately 84 m according to the equation below,

(1) $$d_{max}=\frac{3\times 10^{8}}{2\times (freq_{max}-freq_{min})\times n}\;$$

where n is the number of samples in a ramp up time. Because the target distance of the system for monitoring is closer than 50 m, the reduced sampling rate is sufficient to support the objective.

For real-time displaying of the processed result, all the data processing units perform in parallel. The data processing flow is depicted in Figure 7 (right). In this research, the Arduino board performed as ADC collects one second worth of data and sends them to Raspberry Pi 3, because we assume that 1 second time interval of data collecting is acceptable for the purpose of monitoring a certain area. The time period of the data collection can be adjusted according to the purpose of the use of the system. After the data is collected into the Raspberry Pi 3, the data are published in the RMQ protocol to further data processes. After the data are processed through each postprocessing method, the processed data are sent back to the server inside the Raspberry Pi 3 and ready to be consumed by users. In this research, postprocessing is IFFT, and it takes about a few tens of milliseconds in the Raspberry Pi 3. The postprocessing scripts can be performed in more computation intensive devices to shorten the processing time further.

## 5. Experimental Results

Experimental set-ups for each version of the radar system are different due to the design changes on the system configuration and data processing procedure. As explained in the previous section, a real-time data processing procedure was impossible for the initial version.

### 5.1. Initial Version

**Figure 8:**
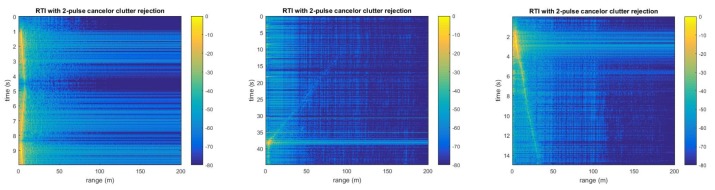
Ranging test results using version zero radar [11].

Experiments for measuring the distance of the object are performed to verify the detection range and accuracy of the radar system [11]. The experiments are configured at a roadway beside of a factory near Purdue University West Lafayette campus. The moving target for the experiments is a medium sized sedan. The radar is installed on an edge of the place and each target object approaches toward the radar from the distance of 100 m. The experiments with the car are performed with two different velocities, and the car passes by the radar while trying to maintain its speed. The velocities of the car for the experiments are 10 mi/h and 15 mi/h, which are approximately 6.25 km/h and 9.4 km/h, respectively. However, the speeds measured by the experiments were not exactly matched with the speeds stated above because the car was driven manually. The data were recorded as audio files and analyzed through a MATLAB script that performs IFFT later. In the experiments, the car was detected in the range of 100 m as shown in the middle of Figure 8. The intensity of the received signal is stronger when the car was moving in close range (Figure 8, left), comparing when the car was moving in distance (Figure 8, middle and right). Figure 8 shows significantly low noise because the adopted commercialized RF components have an optimize maximum SNR and low noise. Moreover, the place where the experiments were performed was quite in terms of electromagnetic fields, thus the radar system has a significant low disturbance.

Experiments with the initial version indoors were also performed to verify the capability of the radars at the in that environment [10]. A building on the campus is chosen for the experiments. A class 1 UAV, DJI Phantom, is employed and the radar is installed at the end of one side of the room. The distance between the radar and the wall placed at the opposite end of the room is ~5 m. The experiments with the drone are performed in two cases: the drone is covered with or without reflection tape on its body. Covering the drone with the reflection tape assists to increase total reflected signals from the drone [10]. The drone is controlled manually and moves back and forth in front of the radar system. Because of the manual control, the speed of the UAV varied and the exact velocity and distance information of the drone are roughly estimated. As like the previous experiments, data processing is performed through the MATLAB script performing IFFT after data are collected into audio files.

**Figure 9:**
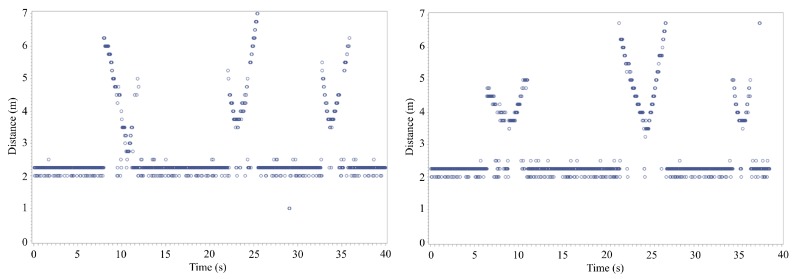
Drone distance measurement experiment results using version one without reflection tape (**left**), and with reflection tape (**right**) [10].

The detection of the drone is shown as clear V-shapes in Figure 9 as the drone moved back and forth [10]. It is difficult to conclude that the maximum distances of the drone in the conference room was different based on applying on or removing reflective tape. Nevertheless, the intensity level of the reflected signals is different. The maximum intensity when the radar detects drone covered with reflection tape is approximately −11 dBm, whereas the maximum intensity without reflection tape is approximately −20 dBm. This means that the reflection power of the drone covered with the reflection tape is ~8 times higher than the case without the reflection tape. However, the intensity can be affected by the relative position of the drone. When a drone increases its speed, the body of the drone is tilted forward so that radar cross section (RCS) of the drone is reduced. It is possible that the power of the reflected signals might have been reduced due to the UAV’s action.

### 5.2. Improved System

**Figure 10:**
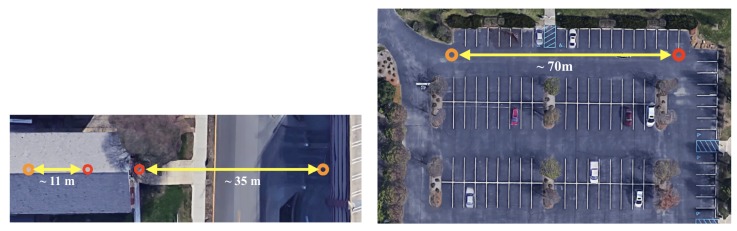
Indoor (left arrow) and outdoor short distant (right arrow) experiment setup (**left**); outdoor experiment set-up (**right**).

To verify the detection range and accuracy of this improved system, experiments to measure the distance of the object are performed as same as the previous version. The experiments are performed at the main floor of a building on campus, in front of the building, and a parking lot near the campus as shown in Figure 10. The target object moves back and forth along the line of the arrows between red and orange circles. Red circles in Figure 10 show the position where the radar was placed when the experiments were performed, and the orange circles show the most distant location of the target from the radar. The target for the experiments in the front of the main floor of the building and in front of the building is a person. Further, the targets for the experiments conducted at the parking lot are a medium sized sedan, a person, and a class 1 UAV–DJI Phantom.

#### 5.2.1. Experiments in Campus

When the experiments are carried out at the main floor of the building, the radar is installed at the one end on the main floor as shown as the left red circle in Figure 10 (left). The person moves back and forth in a range of 11 m, which is the maximum distance that can be reached on the main floor of the building. Moreover, when the experiments are carried out right in front of the building, and the radar is installed at the front of the building as shown as the right red circle in Figure 10 (left), the person moves in front of the radar system at the same as the experiments performed inside the building. The maximum distance that the person moved was approximately 35 m.

Data processing during the experiments is performed in real-time through python scripts to collect data, perform IFFT, and plot the processed data. The data is collected for a second through the ADC board and sent to the Raspberry Pi 3. After the one-second length of data is collected, the data is processed through IFFT. The data processing time for performing IFFT is about a few hundreds of milliseconds in the raspberry pi 3. Last, the processed data is sent to the displaying device to help users track distance of the object in real-time. Throughout the data processing, the delay of the process to visualize collected data for a user takes about 2 seconds.

**Figure 11:**
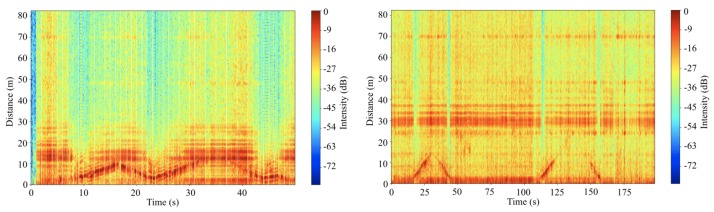
Distance measurement of a person at the main floor of the building (**left**) and in front of the building (**right**).

The results of the experiments are shown in Figure 11. The solid red line at ~13 m in the Figure 11 (left) is the wall facing the radar, and red line moves up and down is the person moving in front of the radar. The results show that when the person gets close to the radar so that the reflection from the person overwhelmed and blocked reflections from walls and other objects, the noise level goes down and the line at about 13 m also disappeared. A similar circumstance is also showed when the experiments are performed at the outside of the building. The solid red line at ~30 m in the Figure 11 (right) is garage wall facing the radar, and red line moves up and down is the person moving in front of the radar. Because the garage wall is more like a collection of poles, reflections from further poles also detected by the radar. Because the intensity of transmission frequency of the radar is 32 times stronger than its early design, the strength of reflected signals are much clearer than the previous experiments. Additionally, the signal reflected from the ground is the strongest signal that the system received in the experiments.

**Figure 12:**
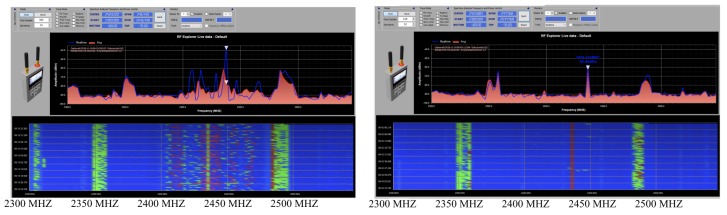
Radio environmental measurement near the building (**left**) and in the park (**right**).

Additionally, the system is also disturbed by the Wi-Fi signals during the experiments. As shown in Figure 12 (left), there are strong signals around 2.3 GHz at −80 dBm, 2.35 GHz at −70 dBm, 2.43 GHz at −65 dBm, and 2.48 GHz at −65 dBm when the experiments are performed inside or outside of the building. Those are identifiable Wi-Fi signals provided in buildings nearby. Furthermore, additional signals are spatially distributed in the range of 2.45 GHz to 2.48 GHz. Those signals can possibly be reflections of the Wi-Fi or other Wi-Fi signals that come from somewhere else. The closed space of the main floor of the building, complex structures of buildings, and landscape around the building limited the experiments by scattering various types of those signals in directions, which can interfere with the system and the signals can be shown as increased background noise. To overcome those limitations, the radar system needs to be installed in an open space in which there are no structures and obstacles except the system and the target in all directions.

#### 5.2.2. Experiments at Open Space

**Figure 13:**
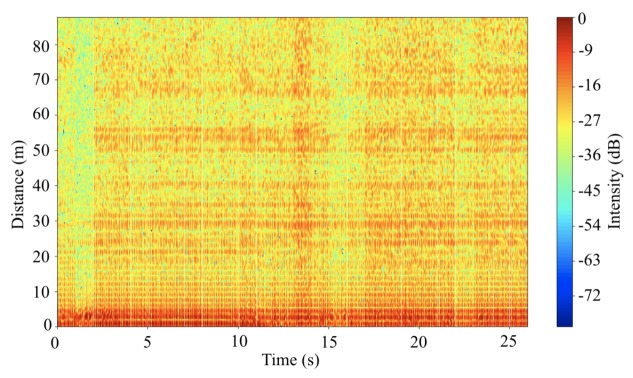
Background noise measured through the radar system at Cumberland park parking lot.

Further experiments are performed to verify the system in an open space. Before the radar system performs for experiments, background noise is measured. The background noise includes clutters. Clutters mean the reflection signals from trees, ground, and other objects located nearby the area where the experiments are performed. Because the antennas of the system are installed 1.5 m above the ground and the surface of the antenna where transmits and receives signal are perpendicular to the ground, the system continuously receives reflected signal from the ground. With regards to the vertical and horizontal beamwidth at half power of the antennas, the distance where the ground reflects the half power is ~1.73 m. Because of the short distance, clutter from the ground is perceived with strong signal intensity within the height of 1.73 m. Additionally, the intensity of the background noise in each distance and each time frame is not always the same as shown in Figure 13. Random noise seems to remain when the average background noise is removed from the measured data. The remained random noise tends to decrease the accuracy of object detection.

**Figure 14:**
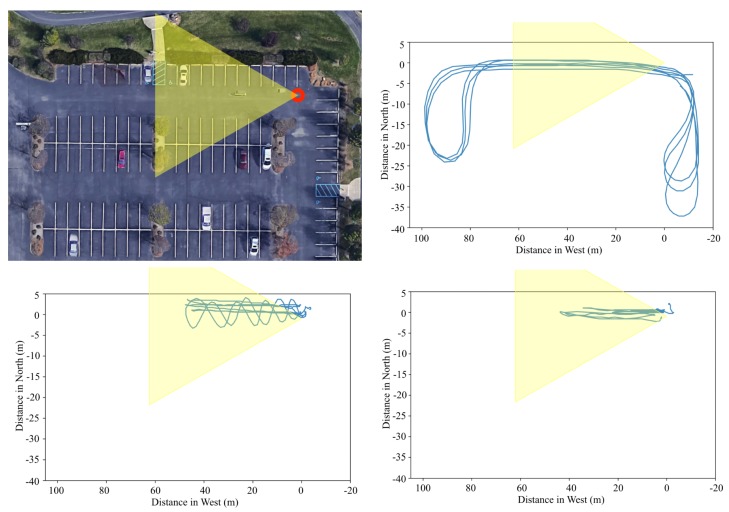
Plot of GPS tracking of each object at the park parking lot; the car, person, and drone in order of top right, bottom left, and bottom right.

Under the known background noise, the radar is installed at a corner of the parking lot as marked as a red circle in Figure 14 (top left). The yellow triangle in the Figure 14 illustrates the field of view of the radar. Targets move back and forth in front of the radar system as shown in Figure 10 (right) as yellow arrows. Moving objects for these experiments are the car, person, and class 1 UAV. The car is driven toward and away from the radar in a range of ~70 m and both the person and the drone move back and forth in a range of ~40 m. The targets are tracked by a commercial GPS module attached to the targets. The geographical movements of the targets are illustrated in Figure 14. The round tails in the car movements shown in Figure 14 (top right) indicate that the car turns around to change its direction. In addition to the back and forth movement, a zigzag motion is performed for the experiment with a person, in order to test if orthogonal movement affects to the detection (See Figure 14 (bottom left)). The drone is taken off and landed in front of the radar system and controlled manually. Nevertheless, we tried to keep the drone moves in a straight line.

The environmental noise in the open space is shown in Figure 12 (right). The system is still disturbed by Wi-Fi signals that are provided at the park and houses near the park. There are strong signals around 2.35 GHz at −65 dBm, 2.44 GHz at −65 dBm, and 2.48 GHz at −65 dBm. However, there is no other considerable signal at the place especially in the operating range of the antenna. The environment is much cleaner than the environment near the building on the campus.

**Figure 15:**
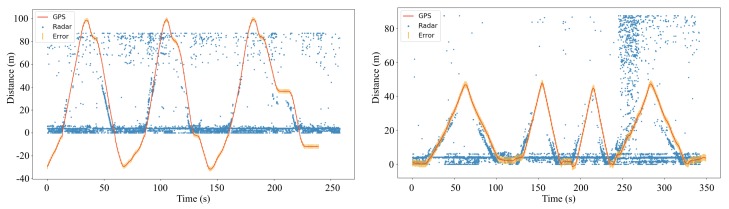
Plots of the movement of the car (**left**) and person (**right**) detected by the radar and also tracked by the GPS at the parking lot.

Figure 15 illustrates the object detection results along with the tracking information obtained from the GPS module that is tied to the object while moving. Blue dots in the figure are the strongest measures at each discrete time, indicating the distance of the object detected by the radar. The red line tracks the GPS-reported movement of the object and is shown as distance from the radar system. The orange line shows an error of 2 m to illustrate the average error bound of the GPS module as the experiment was performed in an open sky area without any obstructive objects (e.g., high-rise building and cloud) nearby. The dense blue dots at a near distance are mostly clutters coming from the ground and tree reflections because the parking lot was surrounded by trees. Further, the blue dots at a far distance are mostly the measures of strong reflections from the trees and other landscape objects around the site (i.e., the cars parked nearby). Despite of the clutters the trajectories of the objects tracked by the radar and GPS are reasonably matched each other, although it is difficult to compare them quantitatively because of the noises and clutters. In general, the error between the GPS tracking and radar detection rises at very near and far distances and becomes lower in the middle. We view it as an issue of detecting only the strongest signal, and the issue can be relieved by adapting advanced filtering algorithms to detect the most likelihood signal towards the object.

**Figure 16:**
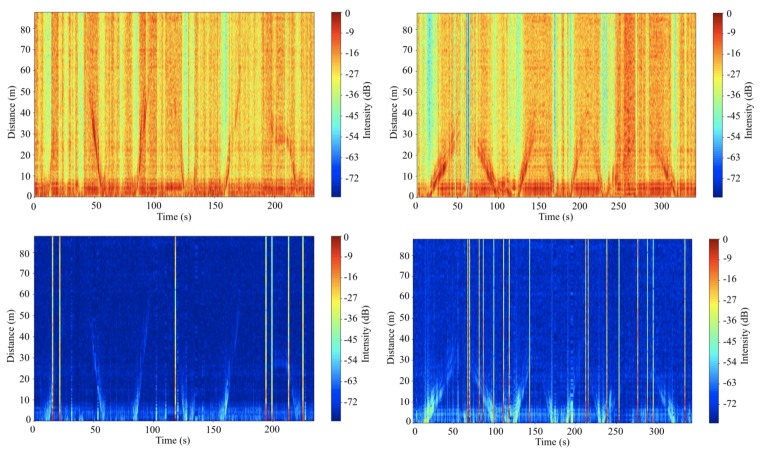
Raw data plot for detection of the car (**top left**) and person (**top right**) and clutter rejected data plot for detection of the car (**down left**) and person (**down right**).

The raw data of object detection results of the car and person are shown in Figure 16. Although discontinuous and significant noise and clutters captured by the radar are shown in the figures, strong reflection signals from the objects are distinguishable. For clear identification of the detection, range–time–intensity clutter rejection method is adopted. The results of the clutter rejection are shown in Figure 16 (bottom). The results show much clearer lines of detection. Broken lines, lighter lines in the figures indicate a bad connection between the system components at the moment. This was simply caused by a bad cable attached to the RF board. Without those broken lines, it seems that the radar can detect the car at ~50 m and the person at ~30 m at maximum. Even if it can detect the object in the range, the noise level needs to be reduced to improve the performance of the radar system for clearer detection.

**Figure 17:**
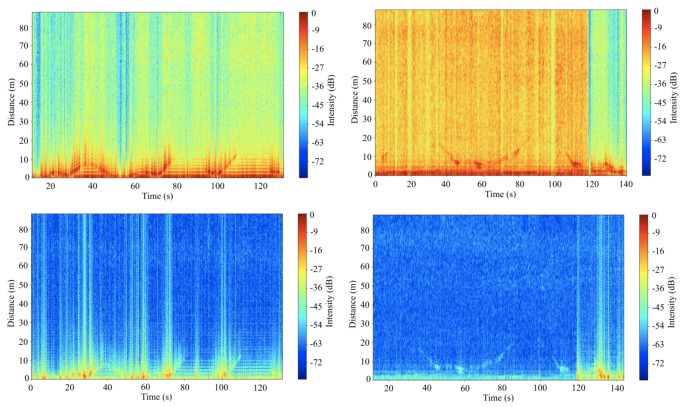
Raw data plot for detection of the drone with reflection tape (**top left**) and without reflection tape (**top right**) and clutter rejected data plot for detection of the drone with reflection tape (**down left**) and without reflection tape (**down right**).

Figure 17 (top) illustrates the drone detection results. The top left image of the Figure 17 shows the experimental result of detection of the drone covered with reflection tape and the top right image shows the experimental result without reflection tape. To illustrate the detection result clearer, the range-time-intensity clutter rejection method is again adopted as shown in Figure 17 (bottom). With regards to the figures, it is difficult to conclude that the maximum detection range of drone covered with reflection tape are further than the drone itself because the small difference in surface area does not affect significantly on the factors. Moreover, the reflection signal power can differ even with the same object in accordance with its attitude. When the flight direction of the drone is changed, the surface area of the drone facing the radar is increased or decreased in time. Thus, the intensities of reflected signals are high when the drone is hovering or moving slowly.

## 6. Conclusions and Future Work

This research presented a compact and cost-effective mobile 2.4 GHz radar system for object detection. The radar system was designed and fabricated to be able to build a system inexpensive and easy to provide accessibility in various research applications. Additionally, the capability of each version of the radar system is verified through the experiments to detect the moving objects targeted in this research.

The experiments were performed at indoor and outdoor places. Strong Wi-Fi signals interfered with the reflected signals even at the outdoor places. Target objects for the experiments were a car, a person, and a class 1 UAV–DJI Phantom. The system was able to detect each of the objects. By maximizing the transmission power of the system to 1 W in the last version, the power of reflected signals seemed stronger with compare to the two earlier versions. The maximum capability of the radar to detect a vehicle is ~50 m, a person is ~30 m, and a drone is ~17 m within the experiments; however, the capability is expected to be improved by reducing the noise of the system through improved hardware, implementing noise-reducing algorithms in the postprocessing phase, and placing the system in location where ground and clutter interference is insignificant.

Although the experiments to detect each object were successful at a certain distance, the results illustrate some limitations in the detection system using the proposed radar. The most important factor that needs to be addressed in the future is the noise of the system. A possible approach to remove the clutter generated by the ground is to increase the angle of each antennas vertical attitude. When the direction of the antennas are tilted horizontally, the propagation pattern of the antennas will result in reduced ground clutter. If the angle between the ground and each of the antennas is 30°, then the intensity of reflection signals from the ground will be reduced lower then half because the vertical beam width at half power of the antenna is 30°, according to the specification of the antenna. However, this method can limit the height at in which moving objects can be detected. By addressing some of the limitations that were discovered throughout the experimental stages of this study, the radar system can be adapted for a large number of task domains such as monitoring, navigation, or tracking objects because of its accessibility.

## Figures and Tables

**Table 1:** Coffee can antenna specification for version zero.

Diameter	100 mm
Length	132 mm
Operating Frequency	2.30–2.40 GHz
(Center Frequency of radar)	(2.35 GHz)
TE11	1.758 GHz
TM01	2.297 GHz
TE21	2.970 GHz
$\lambda_{G}$	179.660 mm
$\lambda_{G}$/4	30.928 mm
λ/4	44.915 mm

**Table 2:** Antenna specification.

Length	356 × 76 mm
Frequency Range	2.3–2.7 GHz
Nominal Gain	10 dBi
Horizontal Beamwidth at 1/2 Power	30°
Vertical Beamwidth at 1/2 Power	30°
Front-to-Back Ratio at 1/2 Power	30 dB
Maximum Power	5 W

**Table 3:** Radar specification.

	Initial	New Version
Transmission Power	25 mW (14 dBm)	1 W (30 dBm)
Operating Frequency	2.30–2.40 GHz	2.30–2.40 GHz
Sampling Rate	44,100 Hz	5862 Hz
Data Processing	Record and process data later	Real-time process
